# Changes in Physical Activity over Time in Young Children: A Longitudinal Study Using Accelerometers

**DOI:** 10.1371/journal.pone.0081567

**Published:** 2013-11-25

**Authors:** Rachael W. Taylor, Sheila M. Williams, Victoria L. Farmer, Barry J. Taylor

**Affiliations:** 1 Edgar National Centre for Diabetes and Obesity Research, Department of Medicine, University of Otago, Dunedin, New Zealand; 2 Department of Preventive and Social Medicine, University of Otago, Dunedin, New Zealand; 3 Department of Women’s and Children’s Health, University of Otago, Dunedin, New Zealand; University of Sao Paulo, Brazil

## Abstract

Previous research has suggested that marked declines in physical activity occur during the preschool years, and across the transition into school. However, longitudinal studies using objective measures of activity have been limited by sample size and length of follow-up. The aims of this study were to determine how overall activity and time in different intensities of activity change in children followed from 3 to 7 years. Children (n = 242) wore Actical accelerometers at 3, 4, 5, 5.5, 6.5 and 7 years of age during all waking and sleeping hours for a minimum of 5 days. Time in sedentary (S), light (L), moderate (M), and vigorous (V) physical activity was determined using available cut points. Data were analyzed using a mixed model and expressed as counts per minute (cpm, overall activity) and the ratio of active time to sedentary time (LMV:S), adjusted for multiple confounders including sex, age, time worn, and weather. At 5 years, physical activity had declined substantially to around half that observed at 3 years. Although starting school was associated with a further short-term (6-month) decline in activity (cpm) in both boys (difference; 95% CI: -98; -149, -46) and girls (-124; -174, -74, both P<0.001), this proved to be relatively transient; activity levels were similar at 6-7 years as they were just prior to starting school. Boys were more physically active than girls as indicated by an overall 12% (95% CI: 2, 22%) higher ratio of active to sedentary time (P = 0.014), but the pattern of this difference did not change from 3 to 7 years. Time worn and weather variables were significant predictors of activity. In conclusion, both boys and girls show a marked decline in activity from 3 to 4 years of age, a decrease that is essentially maintained through to 7 years of age. Factors driving this marked decrease need to be determined to enable the development of targeted interventions.

## Introduction

The health benefits of a physically active lifestyle in childhood are undisputed and include cardiovascular, musculoskeletal, psychosocial and cognitive advantages [1,2]. The physical and psychosocial benefits in younger children are probably less well established, partly due to difficulty in accurately assessing activity in young children. The elucidation of physical activity behaviors and patterns during growth has been enhanced by the advent of accelerometry, which provides an objective and relatively simple tool. This is particularly the case for younger children whose activity tends to be more sporadic and less organized and therefore difficult to assess by parental proxy [1]. 

However, many questions remain regarding how physical activity patterns develop over time in both sexes. This is limited by the cross-sectional nature of most studies [3,4,5], with few longitudinal studies examining changes in physical activity during early childhood. Two recent papers have reported a reduction in physical activity with the transition to school [6,7]. However, it is not clear from this work whether the reduction is permanent or more transitory as both studies only followed up children for 12 months. Little data exists in younger children. A small study in 3-year-old Scottish children reported an increase in physical activity over the same time frame [8]. By contrast, earlier work from our group has shown that considerable reductions in activity are observed during the preschool years, with a marked decline between the ages of 3 to 4 years in both boys and girls [9]. These early reductions in physical activity are concerning given that young children are already spending a significant portion of their day in sedentary activities [10]. Determining why such marked decreases in activity occur and whether they are maintained into the school years is important for identifying periods in life when intervention may be particularly relevant. 

Sexual dimorphism in physical activity is clearly apparent in older children and adolescents [11]. However, the timing of these differences is unknown, with recent reviews in younger children indicating conflicting results [12,13]. These differences presumably arise as a result of widespread variation in sample size, length of assessment, data reduction techniques and the choice of thresholds for determining various intensities of activity of interest [14].

We had the opportunity to examine longitudinal changes in objectively-measured physical activity in early childhood from our Family, Lifestyle, Activity, Movement and Eating (FLAME) study, which collected repeated measures of physical activity in young children at six time-points over a four-year period. The aim of this paper was to examine the changes in physical activity, both overall and by categories of intensity of activity, that occur in boys and girls from preschool (age 3 years) to two years after starting school. 

## Materials and Methods

### Ethics Statement

The study was approved by the Lower South Regional Ethics Committee (Reference OTA/04/03/023). Signed informed consent was obtained from the parents/guardians.

### Participants

FLAME recruited a birth cohort of healthy children just before their third birthday from the only maternity hospital servicing Dunedin, New Zealand (population 120,000). Two hundred and forty-four families participated from an eligible pool of 413 (59% response rate) in the longitudinal study and few differences were observed between participating and non-participating children at baseline [9]. The sample consisted of 107 girls (44%) and 137 boys and children were predominantly Caucasian. 

### Measures

Participants were seen at University research clinics every six months from 3 to 7 years of age. Height was measured in duplicate to the nearest 0.1 cm using an electronic wall-mounted stadiometer (Heightronic, QuickMedical, WA, USA) and weight by electronic scales (Mettler Toledo, USA) in duplicate to the nearest 0.1 kg. Body mass index (BMI) was calculated (kg/m^2^) and BMI-for-age z-scores determined using US reference data [15]. Waist circumference was measured immediately above the iliac crest directly against the skin.

Physical activity was measured at 3, 4, 5, 5.5, 6.5 and 7 years of age using omnidirectional Actical accelerometers (Mini-Mitter, Bend, OR) [16]. The accelerometers were placed on the child’s waist at the clinic and the parents were instructed to keep the monitors on at all times for at least five consecutive days. A commercial data reduction program (MeterPlus version 4.2, Santech Inc, La Jolla, CA) was used to analyze the data. Although MeterPlus is designed for analysis of Actigraph accelerometers rather than Acticals, we developed a computer program (Gavin Kennedy, Fuzzy Systems Ltd) which converted Actical .awc files to .csv files suitable for use in MeterPlus. A test comparison of 10 accelerometer files analyzed using the MeterPlus and the Actical proprietary reduction program showed that MeterPlus produced comparable results and was thus suitable for use with the Actical data. Epoch length was set at 15 seconds to account for the intermittent and sporadic nature of young children’s activity [1]. Data were excluded if the total counts for the day were less than 10,000 or more than 20 million, which may indicate accelerometer malfunction [17]. As our subjects wore the accelerometers for 24 hours a day, we removed sleep time from the analyses so that these data represents waking hours only. Parental sleep diaries were used to determine time to bed and time awake each day. Filters were then created in MeterPlus to exclude data from the time the child went to sleep at night until they arose in the morning. Thus any physical activity during this total sleep period was also excluded, although in practice, this was very minimal. Once sleep was removed, non-wear time was defined as at least 20 consecutive minutes of zero counts [18]. Usable data were defined according to Penpraze et al [19] who reported acceptable (71%) reliability for accelerometer measures obtained for at least 3 hours per day for 5 days. No published cut points were available for determining the amount of time spent in moderate- to-vigorous physical activity (MVPA) that are suitable across our entire age range (3-7 years). Therefore, minutes of sedentary, light, moderate and vigorous activity were calculated according to the Actical cut points of Evenson et al [20] developed in children aged 5 to 8 years.

### Statistics

All analyses were undertaken using Stata Release 12 (College Station, TX: StataCorp LP). Data were analyzed as counts per minute as well as being divided into components; namely the number of minutes of sedentary, light, moderate, and vigorous activity [20]. However, as accelerometers were worn over 24-hour periods, each day represents a closed dataset ie. individual components (sedentary, light, moderate, vigorous, sleep) are not independent because if more minutes can be attributed to one intensity of activity, fewer minutes *must* be attributed to one or more of the other components. As the observation time for each day was different (after removal of sleep at the individual level), the minutes at each level of intensity (components) were expressed as a percentage of the total observation time. Because the sum of the components is 100%, the components must be analyzed in relative terms [21]. Therefore we used the ratio of light, moderate, and vigorous activity (combined) to sedentary activity as our outcome variable (LMV:S) which was log-transformed before analysis [21]. 

Mixed models with observations nested within age, and age nested within participant were used to examine differences between boys and girls over the four year time period for the number of counts per minute and the ln(LMV:S). The data were analyzed using the Stata estimation command xtmixed with a random effect for participant and age. Counts per minute were log transformed before analysis to reduce the association between the variance and the mean [22]. A variable for the time the accelerometer was worn was also included in the models as well as terms for weekend/weekday, maximum and minimum daily temperature and rainfall. Weather data were obtained from the National Institute of Weather and Atmospheric research (www.niwa.co.nz). These models were compared with models that included interaction terms between age and sex to find out if there were different activity patterns across the time period for boys and girls using a likelihood ratio test.

## Results

Of the 242 children present at baseline, 202 remained in the study at 7 years of age (83% retention). However, not all children provided usable accelerometry data according to our inclusion criteria. Table 1 presents the anthropometric and body composition data for those children who had usable accelerometry data at each age; 227 (94%) had usable data at age 3. Comparable figures for the remaining time points were 215 (89%) at 4 years, 207 (86%) at 5 years, 195 (81%) at 5.5 years, 192 (79%) at 6.5 years and 171 children (71%) at 7 years of age. However, children who dropped out of the study or did not have usable data (n = 72) did not differ at baseline (age 3) from those with usable data at age 7 (n = 170), for height (P = 0.905), weight (P = 0.729), BMI (P = 0.699), or maternal BMI (P = 0.991). However, they were more likely to be non-European (P = 0.005, data not shown).

**Table 1:** Characteristics of the study population at each age.

		Age (years)					
		3	4	5	5.5	6.5	7
Girls	n	99	90	88	85	83	66
	Age (years)	3.0 (0.04)	4.0 (0.03)	5.0 (0.03)	5.5 (0.12)	6.5 (0.03)	7.0 (0.03)
	BMI (kg/m^2^)	16.9 (1.4)	16.8 (1.5)	16.4 (1.5)	16.6 (1.6)	16.7 (1.9)	16.9 (2.0)
	BMI z-score	0.76 (0.84)	0.91 (0.82)	0.68 (0.76)	0.77 (0.70)	0.61 (0.73)	0.58 (0.75)
	Waist girth (cm)	50.3 (3.1)	53.1 (3.9)	54.0 (3.8)	55.9 (4.4)	56.5 (4.7)	57.2 (5.4)
Boys	n	128	125	119	110	109	105
	Age (years)	3.0 (0.04)	4.0 (0.04)	5.0 (0.03)	5.5 (0.04)	6.5 (0.04)	7.0 (0.03)
	BMI (kg/m^2^)	17.2 (1.1)	16.8 (1.2)	16.5 (1.2)	16.4 (1.2)	16.4 (1.3)	16.5 (1.4)
	BMI z-score	0.90 (0.81)	0.85 (0.86)	0.70 (0.78)	0.66 (0.74)	0.51 (0.70)	0.49 (0.71)
	Waist girth (cm)	50.2 (2.9)	52.7 (3.1)	53.8 (3.1)	55.1 (3.2)	56.0 (3.2)	56.7 (3.6)

Data are presented as mean (SD) except for n.

In total 242 children (105 girls and 137 boys) provided 7801 days of accelerometer recordings. The means and standard deviations for the time the accelerometer was worn and the total number of counts per minute for boys and girls at each age are shown in Table 2. Adherence to the measurement protocol was excellent and in general, children wore the monitors much longer than the minimum stipulated time, with little variation between individuals. Table 2 also shows the changes in the LMV:S ratio which incorporates light activity with moderate-to-vigorous activity. Children spent considerably more time (ratios of 3.7-4.0) in active pursuits than being sedentary at 3 years of age. However this ratio declined markedly from the age of 4 years onwards, falling to approximately half the values observed at baseline. Following the significant decline from 3 to 4 years of age, values from 4 to 7 years were similar, with the exception of 5.5 years where a further transitory decline was observed (Table 2).

**Table 2:** Accelerometry variables at each age in boys and girls.

		Age (years)					
		3	4	5	5.5	6.5	7
Girls	Number of valid days	5.6**^*a*^** (0.7)	5.9**^*b*^** (0.8)	6.8**^*c*^** (0.5)	6.9**^*c*^** (0.4)	6.8**^*c*^** (0.5)	6.5**^*c*^** (0.9)
	Hours worn/day**^*1*^**	12.0^ab^ (0.8)	12.1^ab^ (0.7)	12.1**^*a*^** (0.7)	12.0^ab^ (0.7)	12.2^ab^ (0.7)	12.2**^*b*^** (0.7)
	Counts per minute	773**^*a*^** (264)	522**^*b*^** (220)	506**^*b*^** (212)	382**^*c*^** (128)	483**^*b*^** (168)	499**^*b*^** (205)
	Ratio of ^2^LMV:S (%)	3.7**^*a*^** (3.1, 5.0)	2.3**^*b*^** (2.1, 2.6)	2.2**^*b*^** (2.0, 2.4)	1.7**^*c*^** (1.5, 1.9)	1.9**^*b*^** (1.6, 2.1)	1.8**^*b*^** (1.6, 2.0)
Boys	Number of valid days	5.7**^*a*^** (0.7)	6.2**^*b*^** (0.8)	6.9**^*c*^** (0.4)	6.9**^*c*^** (0.3)	6.7**^*d*^** (0.6)	6.5**^*d*^** (0.8)
	Hours worn/day**^*1*^**	11.9**^*a*^** (0.9)	12.0^ab^ (0.8)	12.2**^*b*^** (0.8)	12.2**^*b*^** (0.7)	12.4**^*c*^** (0.7)	12.5**^*b*^** (0.8)
	Counts per minute	813 (249)**^*a*^**	532**^*b*^** (200)	542**^*b*^** (244)	444**^*c*^** (165)	544**^*b*^** (245)	554**^*b*^** (230)
	Ratio of ^2^LMV:S (%)	4.0**^*a*^** (3.1, 4.9)	2.4**^*b*^** (2.2, 2.8)	2.3**^*b*^** (2.0, 2.5)	1.8**^*c*^** (1.6, 2.1)	1.9**^*d*^** (1.7, 2.2)	1.8**^*d*^** (1.6, 2.1)

Data are presented as mean (SD) except for the ratio which is presented as median (25^th^, 75^th^ percentiles).

*1* Once sleep and non-wear time have been removed.

*2* Intensity of activity categories calculated from Evenson et al [20].

Values with different superscripts within the same row indicate significant differences (P < 0.05) by age.

**Figure 1:**
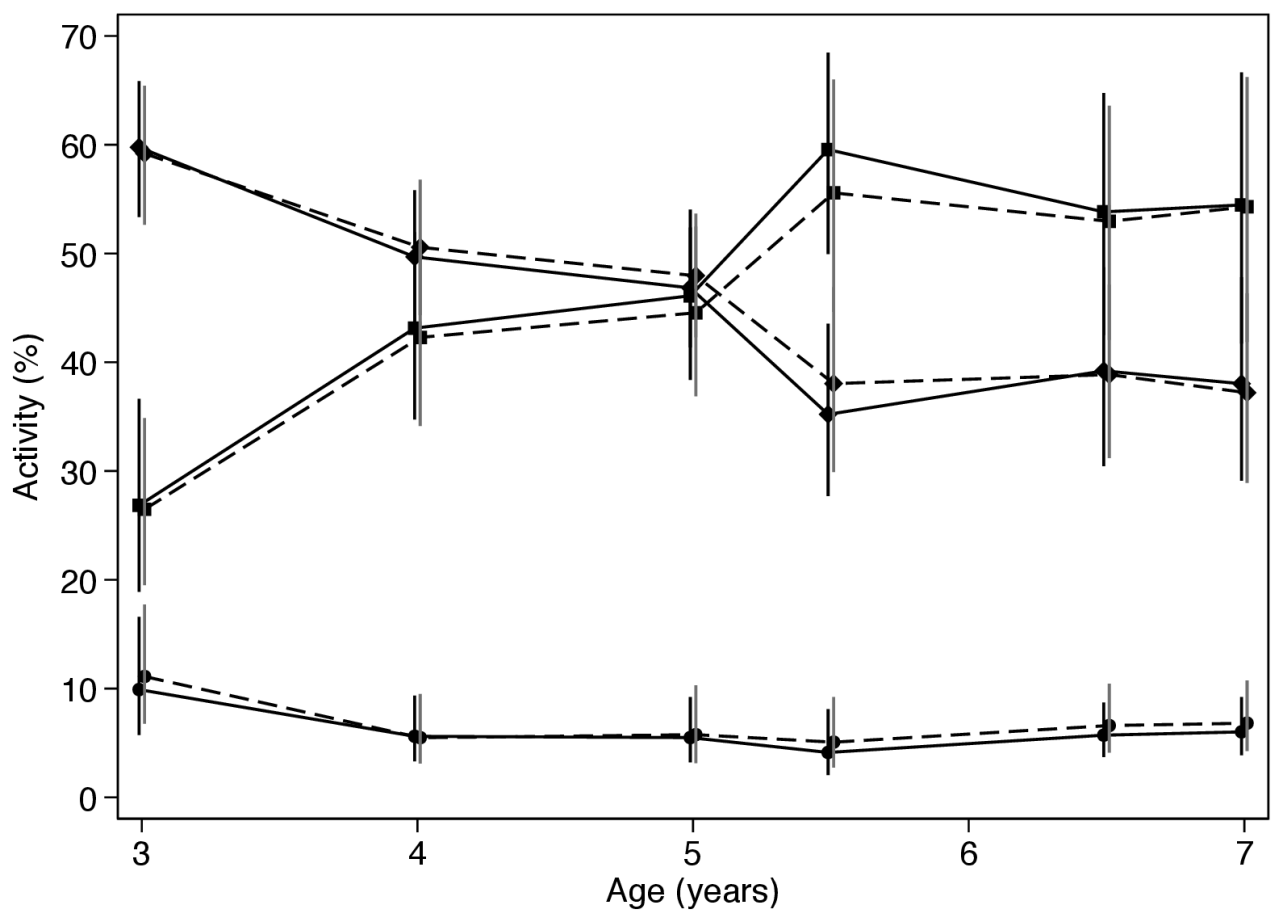
The proportion of time spent in sedentary (■), light (♦), moderate-vigorous (●), activity in boys (solid lines) and girls (dotted lines) from 3 to 7 years of age. Data are unadjusted figures.

**Figure 2:**
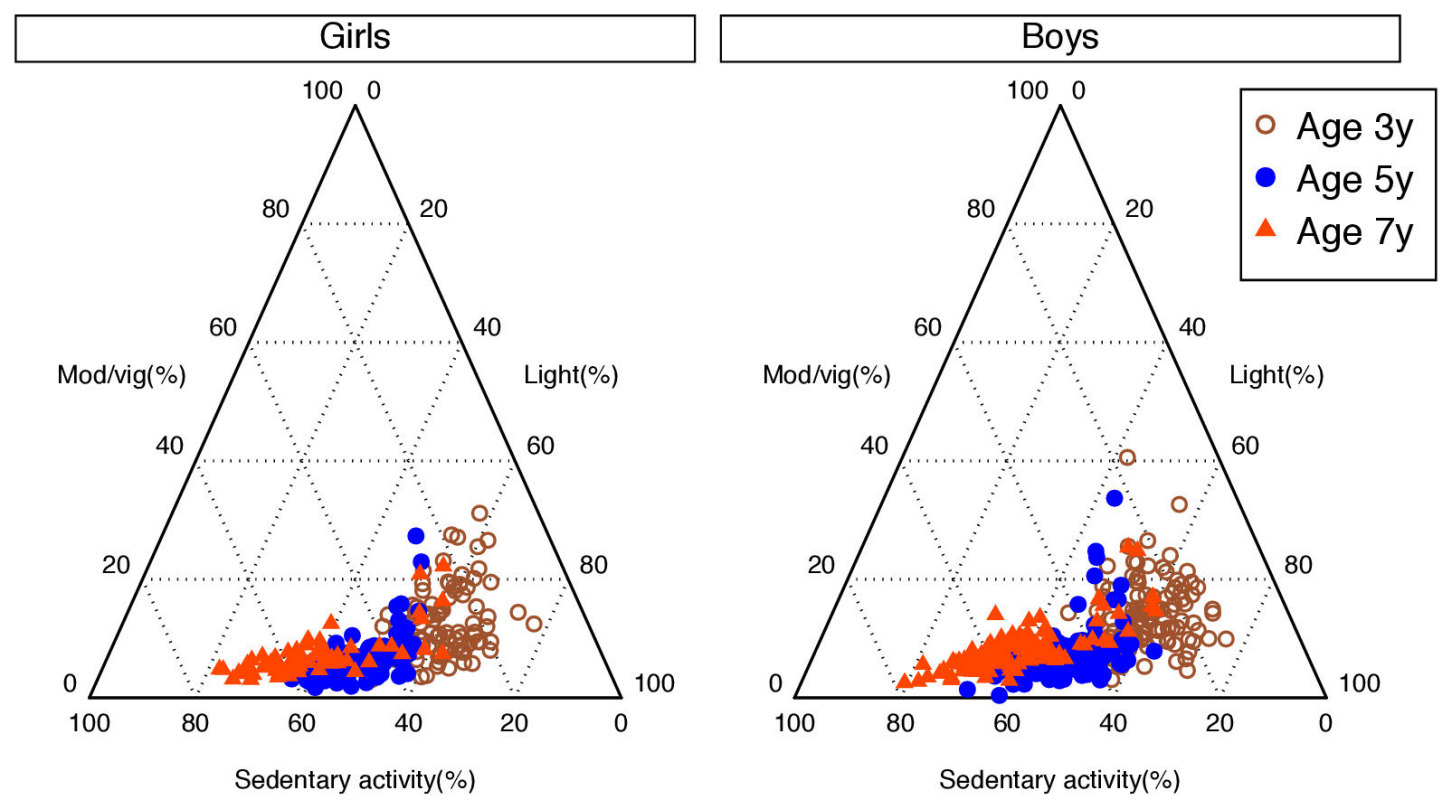
Ternary plot of the individual variability in percentage of time spent in sedentary, light, and moderate-vigorous activity each day at 3, 5 and 7 years of age.

The proportions of each individual type of activity (sedentary, light, moderate-to-vigorous) in boys and girls are shown in Figure 1, with the individual variability highlighted in Figure 2. These figures demonstrate that the marked reduction in the LMV:S ratio occurs as a result of a decline in the proportion of light activity and its replacement by sedentary activity in both sexes as the children’s ages increased. In contrast, differences between girls and boys were small and non-significant.

The parameters for the final models for the predictors of counts per minute and LMV:S are shown in Table 3. These include terms for sex and age, hours worn, weekend and the weather variables. The likelihood-ratio tests comparing these models with those which included an interaction term for age and sex were not statistically significant for either outcome, Likelihood Ratio test = 3.51, P = 0.62 for LMV:S and Likelihood Ratio = 3.89, P = 0.56 for counts per minute (data not shown). In order to interpret the results of this analysis the coefficients have been exponentiated and presented as ratios (95% CI) in Table 3. Thus the ratio of the geometric means for sex (boys versus girls) was 1.12, indicating that the ratio LMV:S is 12% (95% CI 2, 22%) higher in boys compared with girls throughout this period. Thus the pattern of this difference did not change from 3 to 7 years. Sex differences for counts per minute showed a similar pattern but did not reach statistical significance (ratio, 95% CI: 3, -1 to 7, P = 0.078). However, the differences among the age groups were statistically significant (P<0.001). The results also show that that the activity levels declined substantially over the first 2.5 years to a minimum of 41% of 3-year-old levels at 5.5 years of age. Although small increases in light-to-vigorous activity are apparent after this age, they remain considerably lower than those observed at 3 years of age. Inconsistent patterns were observed in terms of weekend and weather variables. Children showed higher counts per minute (3%) but a lower LMV:S ratio (6%) on weekend compared with weekdays. Rain and cold weather were significant dampeners on activity using either measure, lowering activity by 0.3-2% per mm of rain or degree Celsius. Finally, time worn remained a significant predictor of both types of activity measure, with counts per minute and LMV:S being considerably 2-8% higher for each additional hour of observation time.

**Table 3:** Influences on physical activity expressed as counts per minute or the ratio of activity to sedentary time.

	Counts per minute			Ratio of LMV:S activity		
	Ratio	95% CI	P	Ratio	95% CI	P
Sex (boys versus girls)	1.03	0.99, 1.07	0.078	1.12	1.02, 1.22	0.014
Age: 4 (all compared with age 3)	0.62	0.59, 0.64	< 0.001	0.49	0.45, 0.54	< 0.001
5	0.58	0.56, 0.60	< 0.001	0.49	0.45, 0.54	< 0.001
5.5	0.46	0.44, 0.48	< 0.001	0.41	0.37, 0.45	< 0.001
6.5	0.50	0.47, 0.52	< 0.001	0.55	0.50, 0.61	< 0.001
7	0.49	0.47 0.51	< 0.001	0.56	0.51, 0.62	< 0.001
Number of valid hours (hour)	1.02	1.01, 1.03	< 0.001	1.08	1.07, 1.09	< 0.001
Weekend (compared with weekday)	1.03	1.02, 1.04	< 0.001	0.94	0.91, 0.96	< 0.001
Maximum temperature (°C)	0.998	0.996, 0.999	0.002	1.00	0.99, 1.01	0.258
Minimum temperature (°C)	0.997	0.995, 0.999	0.006	0.98	0.97, 0.99	< 0.001
Rainfall (mm)	0.998	0.997, 0.999	< 0.001	0.99	0.98, 0.99	< 0.001

Estimates are expressed as ratios which can also be interpreted as percentage difference

## Discussion

Our results demonstrate that substantial declines in physical activity with a concomitant rise in sedentary time are seen even in very young children followed from 3 to 7 years of age. Although reductions in MVPA are apparent, the increase in sedentary activities predominantly occurs at the cost of activities undertaken at a “light” intensity. Overall, boys were 12% more physically active than girls across this age range, with no difference in the pattern across time. 

Few longitudinal studies have investigated physical activity patterns using objective techniques in this age group and none with multiple repeated measures. Substantial declines [23,24] or no change [25] in activity have been reported over two years in slightly older children. We previously reported that mean activity counts showed a marked decline in this sample population from 3 to 4 years of age, a decrease that was maintained at 5 years of age (just prior to starting school) [9]. We now extend these observations a further two years incorporating the school transition period and including analysis of activity in terms of intensity (sedentary, light, moderate, vigorous). In contrast to our findings, three papers report an increase in activity across 1-2 years in children aged 3-4 years at baseline [8,26,27]. Variation in adjustment for confounders could conceivably contribute to these discrepancies; in our sample, weather and total wear time were all related to physical activity. However, two of these papers include the same group of children measured 12 [8] and 24 [26] months after baseline. Furthermore, all three studies [8,26,27] were very small, involving only 42-63 children, with reasonably poor retention (43-61%) over a shorter follow-up (1-2 years) compared to our larger sample (75% retention over 4 years). The similarity in trajectory of change in boys and girls in our study is consistent with the limited existing literature. Others have reported increases in activity in both boys and girls [8], or non-significant changes in either sex, but a significant difference overall [27]. The remaining study in young children described tracking rather than changes in activity per se [26]. 

The transition to school has been associated with lower activity levels in both cross-sectional [7] and longitudinal analyses [6,28]. Our dataset is unique in following up the children with repeated measures which enables us to show that the decline in activity seen from 5 years (just before starting school) to 5.5 years (full 6 months at school) is a transient phenomenon, perhaps associated with an adjustment to “full-time” schooling. Accelerometry data collected at 6.5 and 7 years of age in these children showed some return to preschool activity levels, albeit not to the significantly higher levels observed when the children were aged 3. Although seasonality has been shown to affect activity levels in children from different latitudes [27,29], the decrease we observed from 5 to 5.5 years was not a function of seasonality as the measurement period for our whole sample spanned a 10-month period, and adjustments were made for temperature and rainfall in the analyses.

What might be influencing this sharp reduction in activity during the preschool years is unknown. Despite a reasonable body of evidence examining correlates of activity in young children, few consistent relationships have been observed [12,13]. Few demographic variables are associated with activity at this age, whereas social/cultural variables including parental interaction with children and group composition, and the physical environment such as play spaces and type of preschool attended, may be important [12,13]. We are unable to comment on whether changes in any of these factors influenced a reduction in activity as no data were collected. However, the age range studied coincides with starting kindergarten in New Zealand, which may explain at least part of the reduction, given that more than 80% of kindergarten time is spent on sedentary activities [4,30].

Although statistically significant, the clinical significance of the sex differences in activity we observed in 3-year old children equate to only 6 minutes a day of moderate-to-vigorous activity. This difference is even smaller at older ages. The differences are also smaller than comparably sized studies, which have reported differences of 8-20 minutes a day in young children [31,32,33]. However, caution should be applied when comparing studies given that different accelerometers and intensity thresholds are not interchangeable and can dramatically affect reported time at different intensities [14]. Although prediction equations have been developed which allow for direct comparisons between studies utilizing different thresholds, these currently apply only to the Actigraph accelerometer [34]. In light of recent reviews illustrating that sex is not a major determinant of physical activity in preschoolers (total or time in MVPA) [13,14], it seems unlikely that the difference of 6 minutes a day observed in our study represents a biologically important difference.

The strengths of our study include a reasonably sized sample that was originally recruited from a birth cohort of children, multiple days of assessment at each time point (average 5-6 days), repeated assessment periods (6 measurement points over 4 years), and controlling for confounders not typically examined (weather and wear time). The wealth of data provided by our study is well illustrated by the number of valid days in this dataset, which number more than 7800, offering a superb opportunity to examine physical activity patterns across this age range. Children had to have at least 3 hours of wear time each day, across a minimum of 5 days to be included in analyses [19]. Although more stringent inclusion criteria have been used such as a minimum of 8- [35] or 10- [36] hours each day, use of these criteria did not affect our results as they would reduce our number of total available days for analysis by only 43 (0.6%) if using an 8 hour minimum or 231 (3.0%) for a 10 hour minimum. The fact that the vast majority of our children wore the accelerometers for considerably longer than the minimum is presumably because our children were wearing the accelerometers 24 hours a day, rather than just during waking hours as is typical. Furthermore, removal for water-based activities including bathing was not necessary, because Actical accelerometers are waterproof. As outlined in our methods, examination of all four components of activity (sedentary, light, moderate, vigorous) requires the use of ratios rather than minutes of activity. Further adjustment for time worn is required to remove potential bias from measurement length [18]. This is highlighted by our findings that time worn remained a significant predictor of activity counts and minutes at different intensities, even once adjusted for other confounding factors. A limitation of our study is the differential loss to follow-up in boys and girls. Overall, 33% of girls dropped out of the study compared with only 18% of boys. However, the main reason for dropping out (> 90%) was leaving the district, rather than choosing not to continue participation. In light of this, it seems reasonable that such drop-outs would not bias our findings.

Given the widespread concern that preschool children spend a considerable portion of their day in sedentary past-times [37], it seems somewhat surprising that our subjects spent at least twice (and up to four times at 3 years of age) as much time in activity as they spent being sedentary (Table 2). This arose for two reasons. Firstly, we included all activity above sedentary (rather than just MVPA) in light of recent recommendations around the world for preschool children to spend at least three hours a day being active (light to vigorous) [38]. Secondly, we chose the cut points of Evenson et al [20] to define intensities of activity in the absence of other Actical cut points spanning the whole age range in our study. However, the Evenson minimum cut point for “light” activity is only 12 counts per 15-second epoch (48 counts/minute), considerably lower than those used in some [39] but not all [40] other studies in young children. Repeating our analyses comparing moderate-to-vigorous activity with sedentary and light combined produced similar analyses and did not change the overall conclusions (data not shown).

In conclusion, the marked decline in physical activity observed in preschool children is essentially maintained for a further two years. What factors might be causing such a marked decline and a corresponding increase in sedentary behavior in such young children is unclear but worrying given general concern about physical activity and inactivity levels in children today. Studies which examine longitudinal changes in activity using objective measures would be strengthened by simultaneous examination of behavioral and lifestyle factors. 
